# Correction: Conjunctivitis, the key clinical characteristic of adult rubella in Japan during two large outbreaks, 2012–2013 and 2018–2019

**DOI:** 10.1371/journal.pone.0272880

**Published:** 2022-08-04

**Authors:** Hidetoshi Nomoto, Masahiro Ishikane, Takato Nakamoto, Masayuki Ohta, Shinichiro Morioka, Kei Yamamoto, Satoshi Kutsuna, Shunsuke Tezuka, Junwa Kunimatsu, Norio Ohmagari

The following information is missing from the Funding statement: This work is supported by research grants from the Emerging/Re-emerging Infectious Diseases Project of Japan, the Japan Agency for Medical Research and Development, AMED (20fk0108095h0002) awarded to SK.
